# Mesenchymal Stem Cell-Derived from Dental Tissues-Related lncRNAs: A New Regulator in Osteogenic Differentiation

**DOI:** 10.1155/2023/4622584

**Published:** 2023-07-04

**Authors:** Yinchun Zhang, Xuan Chen, XiaoXia Yang, Lei Huang, Xiaoling Qiu

**Affiliations:** Department of Endodontics, Stomatological Hospital, School of Stomatology, Southern Medical University, Guangdong 510280, China

## Abstract

Odontogenic stem cells are mesenchymal stem cells (MSCs) with multipotential differentiation potential from different dental tissues. Their osteogenic differentiation is of great significance in bone tissue engineering. In recent years, it has been found that long noncoding RNAs (lncRNAs) participate in regulating the osteoblastic differentiation of stem cells at the epigenetic level, transcriptional level, and posttranscriptional level. We reviewed the existing lncRNA related to the osteogenic differentiation of odontogenic stem cells and emphasized the critical mechanism of lncRNA in the osteogenic differentiation of odontogenic stem cells. These findings are expected to be an important target for promoting osteoblastic differentiation of odontogenic stem cells in bone regeneration therapy with lncRNA.

## 1. Introduction

Many diseases in dentistry induce irreversible bone loss, such as periodontitis, peri-implantitis, and refractory periapical periodontitis. Periodontitis is a chronic inflammatory disease with dental plaque biofilm as the initiating factor. When the inflammation further develops to the deep, it destroys periodontal tissue, including alveolar bone, cementum, and periodontal ligament, eventually leading to teeth loss [1]. After the dental implant is placed, the fibers at the alveolar crest ridge are parallel to the implant but not connected to the implant, which makes it more vulnerable to trauma. When the peri-implant mucosa contacts bacteria, forms of peri-implantitis are similar to periodontitis, resulting in alveolar bone loss [2]. Large-area refractory periapical periodontitis responds poorly to conventional root canal treatment, which can easily lead to treatment failure. Under such circumstances, the bone self-healing capacity of periapical tissue is limited. Complete bone healing cannot be achieved, and even the bone defect will be further aggravated. For these dental inflammatory diseases, conventional treatments aim to control infection and eliminate inflammation, but it is hard to achieve complete self-repair of the bone defect. In addition, craniomaxillofacial bones play an essential role in supporting facial structures and protecting nerves and blood vessels. Inflammatory diseases, trauma, tumor and tumor-like lesions, congenital malformations, and other reasons will cause craniomaxillofacial bone defects, resulting in certain appearance defects and dysfunction in patients.

Bone marrow-derived mesenchymal stem cells (BMSCs) are currently the most widely studied MSCs in bone regeneration. However, due to the invasiveness of extracting BMSCs, researchers have begun to focus on other stem cells with osteogenic differentiation potential [3]. Dental tissue-derived MSCs have the advantages of a wide range of sources, easy access with minimal invasion, and no ethical and moral disputes because they are derived from discarded teeth, better interaction with cytokines and biological scaffolds, and longer cryopreservation [4]. Therefore, odontogenic stem cells have good prospects in replacing BMSCs in the study of osteogenic differentiation.

**Figure 1:**
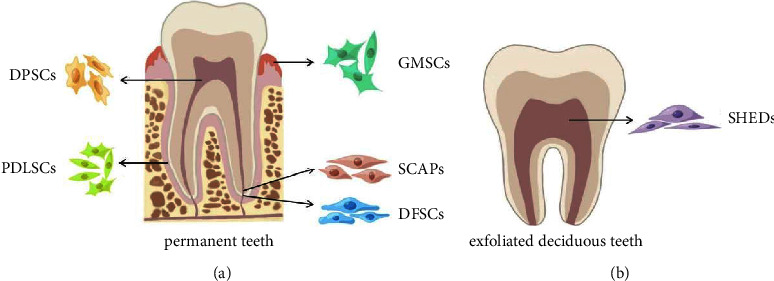
Types of stem cells from dental tissues. (a) Dental stem cells from permanent teeth, including DPSCs, PDLSCs, GMSCs, SCAPs, and DFSCs. (b) Dental stem cells from deciduous teeth mainly SHEDs.

In the oral cavity, different types of odontogenic stem cells have been isolated, including dental pulp stem cells (DPSCs) [5], periodontal ligament stem cells (PDLSCs) [6], gingival mesenchymal stem cells (GMSCs) [7], dental follicle stem cells (DFSCs) [8], stem cells from apical papilla (SCAPs) [9], and stem cells from human exfoliated deciduous teeth (SHEDs) [10] (Figure 1).

lncRNAs are RNAs longer than 200 bp and were initially thought to be unable to encode proteins. However, recent studies have identified a subset of lncRNAs that have the capacity to encode small functional peptides or proteins [11]. Although lncRNAs are present at relatively low levels in cells [12], lncRNAs can regulate gene expression at the transcriptional, post-transcriptional, translational, and epigenetic modification levels by interacting with RNA, DNA, and proteins and then involved in cell growth and metabolism, proliferation, differentiation, and other cellular processes. Increasingly studies have confirmed a significant correlation between lncRNAs and osteo-differentiation of stem cells, which can be used as a regulatory molecule to differentiate stem cells into osteoblasts and influence the process of osteoblasts by affecting the expression of osteogenic markers. Many osteogenesis-related lncRNAs of odontogenic stem cells have been found in recent years. It is reported that lncRNA modification of odontogenic stem cells plays a better role in osteogenic differentiation than cells not transfected with lncRNA. More bone regeneration has been observed in skull defects *in vivo* [13]. Therefore, studying the involvement of lncRNAs in the osteogenesis of odontogenic stem cells is of great significance for bone tissue engineering and can become a new strategy for bone regeneration.

Previously, scholars reviewed the lncRNAs related to the osteogenic differentiation of MSCs and their molecular mechanisms of action [14]. This paper will review the current clinical application of odontogenic stem cells in bone regeneration, summarize and update the osteogenic function of lncRNAs in MSCs, and finally focus on the osteogenic differentiation of odontogenic stem cell-related lncRNAs and their mechanism of action.

## 2. Bone Regeneration Using Dental Tissue-Derived Stem Cells in Clinic

The clinical research on the application of odontogenic stem cells to achieve regeneration and repair of human bone defects is still in its infancy. Only two dental-derived stem cells, DPSCs and PDLSCs, have been reported to be used in the clinical application of bone defect repair.

### 2.1. DPSCs

In 2000, Gronthos et al. first isolated DPSCs from adult dental pulp, which had a similar immunophenotype to BMSCs [5]. The increased expression of osteogenic markers, including alkaline phosphatase (ALP) and type I collagen (COL1), as well as the production of mineralized material, after DPSCs were cultured using an osteogenic medium indicated the ability of DPSCs to differentiate towards osteoblasts [15].

Many clinical trials and case reports of DPSCs in bone regeneration have been reported. Riccardo et al. used autologous DPSCs combined with collagen sponge to form the biocomposite for bone defect repair after extraction of mandibular third molars, and radiographic analyses showed that the mineralization degree at the extraction socket site in the experimental group using DPSCs was significantly higher than that in the group with an only collagen sponge. Histological examination revealed good bone angiogenesis in the group using DPSCs and compact bone formation with lamellar bone arrangement around the Haversian canal. But the control group formed immature bone and had bone resorption [16]. Some researchers transplanted DPSCs into collagen sponge scaffolds and then filled the bone defects of the third molars. Histological analysis and holographic tomography showed that the DPSCs-treated mandible was composed of entirely dense bone, while the non-DPSCs-treated group was established as cancellous bone [17]. Another randomized controlled clinical study using DPSCs to repair alveolar bone loss after tooth extraction did not reach the same conclusion. After autologous DPSCs were incorporated into a resorbable collagen matrix, by comparing the contralateral socket with only a resorbable collagen matrix, the imaging examination after six months found that neither group differed significantly from the other in bone mineral density and bone resorption [18]. The inconsistency in these studies may be due to differences in the extent of bone defects in extraction sockets, the origin and method of isolation of DPSCs, and the duration of follow-up.

In addition to bone defects after the third molar extraction, DPSCs have also been used in regenerative medicine for deep bone defects in periodontitis. In 11 cases of deep periodontal pockets, bone defects were repaired with autologous DPSCs and followed-up for one year. After grafting 12 months, the probing depth (PD) reduction and clinical attachment level (CAL) increase were 5.0 ± 1.3 mm and 4.7 ± 1.5 mm, respectively, compared to baseline. At the 12-month clinical examination, the percentage of probing periodontal pocket sites was 63.6% and 54.5% of the sites CAL gain ≥5 mm [19]. In another randomized controlled study of DPSCs combined with collagen sponge biocomposites implanted into bone defects in chronic periodontitis, the mean PD reduction and mean CAL increase in the DPSCs-treated group were significantly higher than the baseline level after one year. However, the reduction of PD and the increase of CAL in the control group using only collagen sponge had no statistical difference compared with the initial stage. After one year, imaging assessment showed lower intrabony defect depth in the DPSCs-treated group [20].

### 2.2. PDLSCs

Seo et al. first isolated PDLSCs from the periodontal ligament through enzymatic digestion, and the cells expressing STRO-1/CD146 were similar to other MSCs [6]. PDLSCs have higher proliferative capacity than BMSCs, like DPSCs. A subsequent report has demonstrated that PDLSCs possess multipotent MSCs capabilities, as they can differentiate into osteoblasts, chondrocytes, and adipocytes [21]. Furthermore, PDLSCs have the ability to differentiate into cementoblast-like cells, which generate collagen fibers that resemble Sharpey fibers, connecting to cementum-like tissues and contributing to the formation of cementum-periodontal ligament structures in vivo. This suggests that PDLSCs play a crucial role in periodontal regeneration [6]. It is worth noting that cementoblasts and osteoblasts are distinct cell types, and the expression of specific proteins such as cementum attachment protein (CAP) is exclusive to cementoblasts. However, some markers such as ALP and COL1 are shared between cementoblasts and osteoblasts. Compared with other dental stem cells such as DPSCs, SHEDs, and DFSCs, PDLSCs have a higher potential for osteogenic differentiation and bone formation [22, 23]. By constructing experimental models of periodontitis, peri-implantitis, and bone defects, extensive animal experiments have been carried out on the safety and effectiveness of PDLSCs in bone regeneration and periodontal regeneration. Because PDLSCs are derived from the periodontal ligament and are considered one of the most crucial stem cells for the restoration of periodontal tissue, the research on PDLSCs by many researchers mainly focuses on periodontal regeneration. They aim to better realize the repair of periodontal tissue by improving the scaffold materials and growth factors in tissue engineering. The restoration of bone tissue is an integral part of periodontal tissue regeneration.

Due to the ethical limitations of the effectiveness and safety of dental-derived stem cells in clinical applications that have not been fully validated, there are only a few reports on the application of PDLSCs in human stem cell therapy. The earliest clinical trial grafted autologous PDLSCs into the bone defects of 3 male patients with deep periodontal pockets. Three patients' tooth mobility, PD, and CAL significantly improved, and no adverse reactions were seen using this method [24]. PDLSCs obtained from autologous wisdom teeth of 10 patients with periodontitis were made into cell sheets with *β*-tricalcium phosphate granules applied to periodontitis bone defects. The depth of the periodontal pocket, degree of attachment loss, and the height of bone were significantly improved, with no serious adverse events occurring within a mean follow-up of 55 ± 19 months [25]. In a clinical randomized controlled study of bone defects in patients with periodontitis, the experimental group used autologous PDLSCs combined with guided tissue regeneration (GTR) and Bio-Oss. In contrast, the control group only used GTR and Bio-Oss. There was no statistical difference in PD, CAL, and gingival recession between the two groups. Prominent bone regeneration can be observed by X-ray over time, and no noticeable adverse reactions were observed, indicating the safety of PDLSCs sheets for periodontal defects [26]. In another randomized controlled study, PDLSCs and its niche were mixed with gelatin sponges and transplanted into the periodontal defect after flapping as the experimental group, while the control group only performed flapping. Compared with the control group, the bone mineral density of the experimental group in the bone defect area was significantly increased by radiological examination [27]. Among these clinical studies, only the research by Vandana and Shalini showed the superiority of PDLSCs for stem cell therapy in humans [27]. However, the number of samples from these studies was small, and it is necessary to increase the sample size to demonstrate the reliability of their research results.

## 3. lncRNA-Based Scaffolds for Bone Tissue Engineering

Over the past few decades, RNA-based scaffolds have exhibited considerable potential for osteogenesis. Recently, as research on lncRNAs has intensified, numerous studies have reported the development of lncRNA-based scaffolds for bone tissue engineering, aimed at enhancing osteogenic outcomes and biological properties. The formation of strong bone bonding on the surface of titanium implants is a crucial factor for their successful implantation. Recent research has demonstrated the potential of lncRNA hypoxia-inducible factor 1alpha-antisense RNA 1(HIF1A-AS1) to promote the formation of new bone by BMSCs on the surface of titanium implants [28]. A comparable outcome was observed in a separate investigation, where microarray analysis demonstrated an elevation in the expression of lncRNA Prader–Willi region noncoding RNA 1–209 (PWRN1-209) on sandblasted acid-etched titanium surfaces in contrast to the polished titanium surface. Additionally, it validated the upregulation of lncRNA PWRN1-209 augmented the osteogenic differentiation of BMSCs on sandblasted acid-etched titanium scaffold surfaces [29]. The competitive binding of miR-138-5p with bone morphogenetic protein type II receptor (BMPR2) in magnesium-based implants facilitates the promotion of osteogenic differentiation of BMSCs by lncRNA LOC103691336, as evidenced by recent research [30]. The nanostructure of the nanofiber scaffold has the ability to modulate the osteogenic differentiation of stem cells through intricate interactions with lncRNA present in the stem cells. The organized arrangement of aligned nanofibers within the electrospun poly (L-lactide) PLLA scaffold can modulate the osteogenic differentiation of adipose stem cells (ASCs) by regulating the expression of lncRNA H19, which in turn affects the bone morphogenetic protein (BMP) signaling pathway [31]. lncRNA maternal expression gene 3 (MEG3) can inhibit the osteogenic differentiation of BMSCs. By combining lncRNA MEG3 knockdown BMSCs with poly (3-hydroxybutyrate-co-3-hydroxyhexanoate, PHBHHx)-mesoporous bioactive glass (PHMG) and transplanting it to the skull defect of rats, the repair of the defect bone is accelerated [32]. The above study modified the osteogenic ability of bone by combining lncRNA with scaffolds. Despite the nascent nature of incorporating lncRNAs into bone tissue engineering scaffolds, this investigation serves as a benchmark for broadening the utilization of lncRNA in bone tissue engineering and advancing bone regeneration in the forthcoming years.

## 4. Mechanism of lncRNAs Regulating Osteo-Differentiation of MSCs

**Figure 2:**
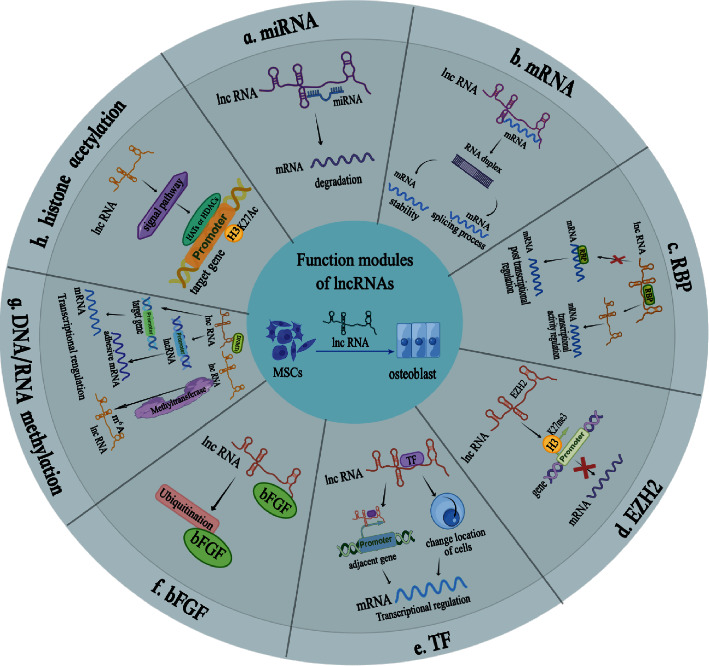
The function module of lncRNA in osteogenic differentiation of MSCs: (a) interaction with miRNA, (b) interaction with mRNA, (c) interaction with RBP, (d) interaction with EZH2, (e) interaction with TF, (f) interaction with bFGF, (g) DNA/RNA methylation, and (h) histone acetylation by Figdraw (http://www.figdraw.com).

In the field of bone tissue engineering, a crucial research direction is the promotion of osteogenesis in stem cells. Recent studies have increasingly demonstrated a noteworthy correlation between lncRNAs and osteogenic differentiation of stem cells, and lncRNAs can affect osteogenic differentiation of stem cells through different mechanisms, including traditional competitive binding of micro-RNAs (miRNAs), direct combination with mRNA, and interaction with RNA binding protein (RBP). In the following, we will provide a comprehensive summary of the current mechanisms by which lncRNAs regulate the osteogenic differentiation of MSCs (Figure 2).

### 4.1. miRNA

miRNAs are a class of endogenous noncoding single-stranded RNAs, and their sequences have approximately 22 nucleotides in length. They negatively regulate target genes mainly. The target mRNA of miRNA contains miRNA response element (MRE). After miRNA combines with the MRE of mRNA, it can degrade mRNA. The activity of miRNA may be affected by the presence of competitive endogenous RNAs (ceRNAs). These ceRNAs can compete with miRNA to bind MREs, and lncRNA can be used as the ceRNA of miRNA. When lncRNA and mRNA sequences are highly homologous, lncRNA can combine with miRNA, acting as a molecular sponge for lncRNA, isolating it from target mRNA, and inhibiting the degradation of miRNA to target genes, which is a classic way for lncRNA to perform biological functions, and it has been widely studied in stem cell osteoblastic differentiation [33] (Figure 2(A)). As an illustration, lncRNA XIXT has been shown to facilitate the expression of runt-related transcription factor 2(RUNX2) through sponge miR-30a-5p positive regulation of osteogenic differentiation [34]. Similarly, lncRNA KCNQ1 opposite strand/antisense transcript 1 (KCNQ1OT1) can regulate BMP2 expression through competitive adsorption of miR-214 to promote osteogenic differentiation [35]. There have been relevant reviews on the molecules involved in the interaction between lncRNA and miRNA to regulate the osteogenic differentiation of MSCs [36].

### 4.2. mRNA

lncRNA can perform functions in the osteo-differentiation of MSCs by directly binding to mRNA. First, lncRNAs play a regulatory role by binding to mRNA and can form RNA duplexes through complementary pairing with mRNA, affecting the stability of mRNA and thus altering protein expression. lncRNA AC132217.4 is highly increased during osteogenic induction of BMSCs. Mechanically found that AC132217.4 binding sites are located on insulin-like growth factor 2 (IGF2) mRNA. The combination can improve the stability of IGF2 expression, thereby activating downstream AKT signal transduction to enhance osteogenic differentiation [37]. In addition, the RNA double-stranded body formed by lncRNA and mRNA also interferes with the splicing process of mRNA and then emerges different splicing forms to regulate the gene expression level. In multiple myeloma (MM), exosomes transport lncRNA-RUNX2-AS1 derived from RUNX2 antisense chains from MM cells to MSCs. RUNX2-AS1 can form double-stranded RNA with RUNX2 precursor, then reduce the expression of RUNX2, and inhibit the osteogenic potential of MSCs by interfering with its splicing [38] (Figure 2(B)).

### 4.3. RBP

RBP contains an RNA domain, which can combine with mRNA to affect mRNA splicing, stabilization, and translation. When RBP binds to lncRNA, RBP cannot bind to mRNA, thus affecting translation to play its corresponding biological function. There are specific bindings between lncRNA taurine up-regulated 1 (TUG1) and RBP Lin28 homolog A(Lin28A), which promotes the osteogenic differentiation of PDLSCs [39]. lncRNA MEG3 and BMP2 competitively bind RBP heteroribonucleoprotein I (hnRNPI), which inhibits the osteogenesis of PDLSCs by affecting the expression of BMP2 [40]. The regulation of lncRNA binding to RBP on the stability of mRNA has also been confirmed in the process of MSCs osteogenesis. RBP TATA box binding protein associated factor 15 (TAF15) is involved in RNA precession and significantly stabilizes RNA. The interaction of lncRNA HOXA transcript at the distal tip (HOTTIP) and TAF15 stabilizes distal-less homeobox 2 (DLX2) and upregulates the expression of osteogenic-related genes after osteoinduction of BMSCs [41]. On the contrary, RBP plays a significant role in acting lncRNA. RBP can affect the stability, location, and transcription of lncRNA in various tumors [42]. Heteronuclear ribonucleoprotein K (hnRNPK) is also a kind of RBP. lncRNA osteogenesis-associated (lncRNA OG) can interact with hnRNPK, acetylate histone H3 lysine 27 (H3K27Ac) of lncRNA OG promoter to increase transcription activity, and then activate and regulate BMP signal pathway to play a positive role in osteogenic differentiation in BMSCs [43] (Figure 2(C)).

### 4.4. Transcription Factors (TF)

LncRNA can also combine with TF to regulate the transcriptional expression of multiple proximal or distal genes. In osteoinduction in MSCs, TF can specifically bind to lncRNA and regulate the expression of target genes at the transcriptional level by affecting TF recruitment to adjacent gene promoters. SRY-box transcription factor 2 (SOX2) is a member of the SOX family. lncRNA MEG3 can separate and bind SOX2 from the BMP4 promoter, then block the activation of SOX2 on the promoter, and reduce the inhibition of SOX2 on BMP4 transcription, to promote osteogenesis of MSCs in patients with MM [44]. In most cases, TFs are located in the nucleus, but when lncRNA binds to specific proteins, the cellular location of the protein can be changed. The interaction between lncRNA HOTTIP and WD40 repeat domain protein 5 (WDR5) (a TF of the WD40 protein family, binding to the *β*-catenin promoter) promotes the translocation of WDR5 to the nucleus and increases the transcription of *β*-catenin in turn. The Wnt/*β*-catenin signaling pathway is activated, thereby enhancing the osteo-differentiation of BMSCs [45] (Figure 2(D)).

### 4.5. Enhancer of Zeste Homologue 2 (EZH2)

LncRNA also interacts with EZH2, a core component of polycomb repressive complex 2 (PRC2). It is the most important subunit with methyltransferase catalytic activity. It can regulate gene expression by epigenetic modification, for example, histone methylation. In osteoblastic differentiation of stem cells, trimethylation at lysine 27 of histone H3 (H3K27me3) is mainly used to bind histones with this modification to the promoter region of the target gene to inhibit the expression of the gene. lncRNA HOXA cluster antisense RNA 3 (HoxA-AS3) interacts with EZH2 to change the level of H3K27me3 in the promoter region of RUNX2 and inhibits the transcription of RUNX2, which regulates the osteogenic induction of MSCs negatively [46]. lncRNA ANCR can also bind to EZH2 and inhibit osteoblast differentiation by downregulating the expression of RUNX2 [47] (Figure 2(E)).

### 4.6. DNA/RNA Methylation

DNA methylation and RNA methylation are frequently observed epigenetic modifications. Specifically, a significant association exists between lncRNA and DNA methylation. DNA methylation is characterized by the covalent bonding of a methyl group to the fifth carbon of cytosine in CpG dinucleotide with a methyl group [48]. Research indicates that lncRNA can regulate DNA methylation or demethylation by interacting with DNA Methyltransferase (DNMT) or ten-eleven translocation (TET), thereby influencing gene expression [49]. DNA methylation can also regulate the expression level of lncRNA. When the promoter region of lncRNA is at a high methylation level, it usually inhibits the transcription of lncRNA to affect gene expression [50]. The osteogenic potential of adipose stem cells from diabetic osteoporosis patients (DOP-ASCs) is lower than that of normal adipose stem cells. Studies have found that inhibiting the osteogenic potential of ASCs in the diabetic osteoporosis population is related to lncRNA-AK137033. AK137033 silencing can inhibit the osteogenic capacity of DOP-ASCs by reducing the DNA methylation level in the promoter region of secreted frizzled-related protein 2 (SFRP2), an antagonist of the classical Wnt pathway. When AK137033 is overexpressed, ASCs have been saved from low osteo-differentiation levels caused by DOP. Therefore, it is possible to explore the correlation and mechanism of lncRNA and DNA methylation in stem cell osteogenic differentiation to improve the osteogenic differentiation potential of stem cells [51]. The osteogenic differentiation ability of BMSCs in patients with aplastic anemia (AA) is weakened. Compared with regular patients, lncRNA MEG3 is low, while the expression of DNMT1 is high. Further studies found that DNMT1 can inhibit the osteoinduction of BMSCs by mediating the hypermethylation of the MEG3 promoter, making MEG3 expression down-regulated, and inhibiting BMP4 transcriptional activity [52]. However, there are no studies on the mechanism of lncRNAs regulating the osteogenic differentiation of MSCs after interacting with TET.

The process of RNA methylation in lncRNA entails the introduction of methyl groups to the nucleotides of RNA molecules, thereby influencing the stability, local conformation, and functional performance of lncRNA. At present, various common lncRNA methylation modifications have been identified, such as N6-methyladenine (m^6^A), 5-methylcytosine (m5C), and 2′-O-methylnucleotides (Nm) [53, 54]. One of the prevalent forms of RNA methylation in long noncoding RNA (lncRNA) is m^6^A (N6-methyladenosine) methylation, which involves the methylation of the 6th position *N* of RNA adenosine (*A*) and can affect the stability and expression of lncRNA [55]. RNA methylation of lncRNA can impact the stability and expression of lncRNA. The methylation of lncRNA has been shown to influence the osteogenic differentiation of stem cells, with methyltransferase-like 3 (METTL3) being an RNA methyltransferase that can enhance the osteogenic differentiation of ASCs by regulating the m6A of lncRNA RP11–44 N12.5 [56] (Figure 2(F)).

### 4.7. Histone Acetylation

Histone acetylation is achieved by acetylation or deacetylation of histone lysine residues catalyzed by histone acetyltransferase (HAT) or histone deacetylase (HDAC), thereby promoting or inhibiting gene transcription. Histone acetylation can dissociate DNA and histones, loosen nucleosome structure, and specifically combine transcription factors with DNA to improve gene expression. lncRNA NKILA is a positive regulator during the osteoinduction of MSCs. NKILA has the capability to impede NF-*κ*B, diminish the enlistment of HDACs through the alteration of NF-*κ*B's binding to the RUNX2 promoter, and decrease the transcriptional repression of RUNX2 by diminishing the deacetylation of H3K27 [57]. In the process of osteogenesis induction, lncRNA HOTAIRM1 can activate NK/AP-1 signal transduction and improve the activity of c-jun, an essential factor of the AP-1 family. C-jun can recruit p300 (a kind of HAT) to the promoter of RUNX2, promote H3K27Ac, and promote RUNX2 transcription to surface modification level [58] (Figure 2(G)).

### 4.8. CXC Chemokine Ligand-13 (CXCL13)

Some researchers found that CXCL13 has a nonnegligible role in the osteo-differentiation of MSCs. A high glucose environment inhibited the differentiation of BMSCs into osteoblasts through decreased expression of lncRNA AK028326 and CXCL13. When lncRNA AK028326 was overexpressed, it could induce the restoration of the osteogenic capacity of BMSCs by increasing the expression of CXCL13 [59] (Figure 2(H)).

## 5. lncRNAs That Regulate Osteogenic Differentiation of DPSCs

**Figure 3:**
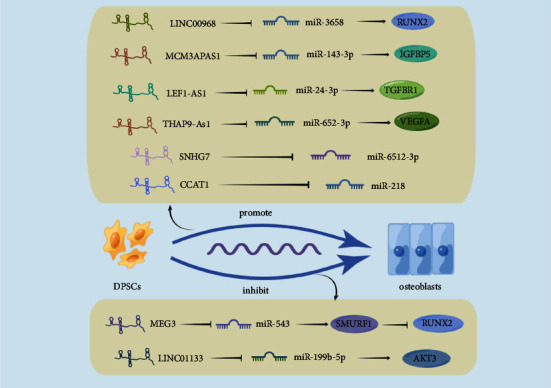
Mechanism of lncRNAs regulating osteogenic differentiation of DPSCs by Figdraw (http://www.figdraw.com).

DPSCs are a type of stem cell that originate from the dental pulp and possess the ability to self-renew and differentiate into various cell types, including osteoblasts. The expression of lncRNAs changed during the osteoinduction stage of DPSCs. Liu et al. induced osteogenesis of DPSCs after 14 days. 89 lncRNAs were differentially expressed, 28 up-regulated and 61 down-regulated [60]. In another study, osteogenic differentiation of DPSCs after TNF-*α* induction, differentially expressed lncRNAs were detected by RT-PCR after 7 days, and there were 77 (58 increased and 19 decreased) and 133 (73 increased and 60 decreased) differentially expressed lncRNAs, respectively after 14 days of treatment [61]. The osteogenesis mechanism of DPSCs regulated by lncRNA can be seen in Figure 3.

### 5.1. The Regulatory Role of LINC00968 in the Osteogenic Differentiation of DPSCs

LINC00968 is located on chromosome 8q12.1. It is a newly discovered lncRNA located in the gene interval in recent years, which is considered related to tumor progression. It has been found to play an important role in the studies of lung adenocarcinoma [62, 63], osteosarcoma [64], ovarian cancer [65], and other diseases. The microarray analysis of Liao et al. revealed that LINC00968 expression significantly escalated during the osteoinduction of DPSCs stimulated by BMP-2. LINC00968 acts as a molecular sponge of miR-3658. LINC00968 promoted BMP-2-induced osteogenic differentiation of DPSCs, while miR-3658 repressed this process. There are binding sites between miR-3658 and RUNX2's 3′UTR, and LINC00968 can inhibit the degradation of RUNX2 by miR-3658 by acting as a competitive endogenous RNA. As we know, RUNX2 is an osteogenesis-specific transcriptional regulator, and LINC00968 affects the osteogenic differentiation of DPSCs in the above way. As we know, RUNX2 is an osteogenesis-specific transcriptional regulator, and LINC00968 affects the osteogenic differentiation of DPSCs in the above way. Then, by transplanting DPSCs transfected with LINC00968 and cotransfected with LINC00968 and miR-3658 into bone defects in immunodeficient mice, respectively, they found that LINC00968 could promote ectopic bone formation, but this process could be reversed by miR-3658*in vivo* [66]. However, relevant rescue experiments to illustrate the effect on cellular osteogenic function were lacking when verifying the ceRNA mechanism in this study, although they have added it in animal experiments.

### 5.2. The Regulatory Role of lncRNA LEF1-AS1 in the Osteogenic Differentiation of DPSCs

LEF1 antisense RNA 1 (LEF1-AS1) is encoded at the lymphoid enhancer-binding factor 1 (LEF1) site. It is an antisense lncRNA located on chromosome 4q25. It is up-regulated in the proliferation or metastasis of ovarian cancer [67], prostate cancer [68], nonsmall cell lung cancer [69], and other tumors. Regarding affecting the differentiation of stem cells into osteogenic cells, research showed that LEF1-AS1 could promote the osteoinduction of DPSCs via sponging miR-24-3p. There are direct binding sites between lncRNA LEF1-AS1 and miR-24-3p. And TGFBR1 (TGF-*β* receptor 1) can specifically bind to miR-24-3p. LEF1-AS1 regulates the expression of TGFBR1 through miR-24-3p in osteo-differentiation of DPSCs [70]. TGFBR1 has a certain significance in osteogenic differentiation. Some scholars have found that the osteogenic differentiation of tooth germ mesenchymal cells can be alleviated by inhibiting TGFBR1, which shows that TGFBR1 is an important target for regulating the osteogenic differentiation of odontogenic stem cells [71].

### 5.3. The Regulatory Role of lncRNA MCM3AP-As1 in the Osteogenic Differentiation of DPSCs

MCM3AP Antisense RNA 1 (MCM3AP-AS1) maps to chromosome 21q22.3. It has been reported that MCM3AP-AS1 enhances chondrocyte viability [72]. Regarding regulating osteogenic differentiation, MCM3AP-AS1 is a sponge for miR-143-3p to promote osteo-differentiation of DPSCs, and miR-143-3p directly binds to the insulin-like growth factor binding protein 5 (IGFBP5) in DPSCs, which can target the expression of IGFBP5. The rescue test found that the promotion of MCM3AP-AS1 overexpression on osteoinduction of DPSCs could be inhibited by silencing the expression of IGFBP5 [73].

### 5.4. The Regulatory Role of lncRNA THAP9-As1 in the Osteogenic Differentiation of DPSCs

Trihydroxyacetophenone domain containing nine antisense RNA 1 (THAP9-AS1) is located on chromosome 4q21.22 and has been observed in some cancers. It can promote the growth of pancreatic ductal adenocarcinoma cells by enhancing the expression of Yes-Associated Protein (YAP) [74]. YAP is an effector that activates the Hippo signaling pathway. Studies have shown that YAP is an important effective protein that regulates the balance of osteogenic-adipogenic differentiation of MSCs. Up-regulation of the expression of YAP can boost the osteogenic differentiation of MSCs and inhibit adipogenic differentiation [75], and YAP can also promote the osteogenic differentiation of PDLSCs *in vitro* [76]. Therefore, some scholars have researched the crucial role of lncRNA THAP9-AS1 in the osteo-differentiation of DPSCs. It was found that the expression of THAP9-AS1 gradually increased in this process. Knockdown of the lncRNA would reduce the expression of early osteogenic markers, including ALP, RUNX2, and COLI. Among miRNAs with potential THAP9-AS1 binding sites, miR-652-3p is the most significant change in response to silent THAP9-AS1. Vascular endothelial growth factor A (VEGFA) is the direct target of miR-652-3p, and its overexpression reverses the inhibition of THAP9-AS1 knockdown on osteogenic differentiation in DPSCs [77]. VEGFA is an important growth factor and the coordinator of the coupling of osteogenesis and angiogenesis. Many studies have found that VEGFA mediates the osteogenic differentiation and bone formation of stem cells [78, 79].

### 5.5. The Regulatory Role of lncRNA SNHG7 in the Osteogenic Differentiation of DPSCs

Inflammation can also affect the mineralization and tissue regeneration ability of DPSCs. lncRNA Small nucleolar RNA host gene 7 (SNHG7) can affect the osteo-differentiation of DPSCs. The expression of SNHG7 increased during the process, silencing the expression of SNHG7 suppressed the osteo-differentiation of DPSCs [60]. Chen et al. treated DPSCs with TNF-*α* to simulate the inflammatory environment. The results showed that under the high concentration (50 ng/mL) TNF-*α* treatment, the expression of SNHG7 gradually decreased during the osteogenic differentiation of DPSCs. However, overexpression of SNHG7 could reverse the inhibition of calcium deposition by 50 ng/mL TNF-*α*. Further experiments found that SNHG7 sponge miR-6512-3p and miR-6512-3p could also reverse the effect of SNHG7 on the osteogenic differentiation of DPSCs treated by TNF-*α*. It is confirmed that in the inflammatory environment induced by TNF-*α*, SNHG7 promotes the osteogenesis of DPSCs by inhibiting the expression of miR-6512-3p [80]. However, the target gene of miR-6512-3p regulating the osteogenic differentiation of DPSCs is unclear and needs further research. The above-related studies, whether in an inflammatory environment or not, have shown that lncRNA SNHG7 can promote osteogenic differentiation of DPSCs. The repair of bone defects usually occurs under inflammatory conditions, but most of the current studies were conducted based on noninflammatory conditions, and it is more relevant to simulate an inflammatory microenvironment to study the mechanism of lncRNA regulation of osteogenic differentiation of dental-derived stem cells.

### 5.6. The Regulatory Role of lncRNA MEG3 in the Osteogenic Differentiation of DPSCs

LncRNA MEG3 is located on human chromosome 14q32.3, with a length of 1595 bp, and is the first lncRNA found to inhibit the growth of tumor cells [81]. It restrains the proliferation and growth of gastric cancer [82], prostate cancer [83], endometrial cancer [84], and other tumors, so it is considered a new tumor suppressor. lncRNA MEG3 may have different results in its regulatory effects on different MSCs. MEG3 and BMP4 are both located on chromosome 14q. It was found that MEG3 can inhibit the transcriptional activity of SOX2 by separating SOX2 from the BMP4 promoter to activate the transcriptional activity of BMP4 to promote the osteogenesis process of BMSCs [44]. Zhao et al. found that it can also play a regulatory role in the osteogenesis of DPSCs. However, unlike the promoting role it plays in BMSCs, MEG3 was down-regulated progressively during osteogenesis induction. And the expression of RUNX2 was negatively correlated with MEG3. Research on its mechanism, miR-543 is the expected target site of lncRNA MEG3 and Smad ubiquitylation regulatory factor-1 (SMURF1) [85]. SMURF1 targets RUNX2 for degradation, thereby inhibiting osteogenic differentiation [86]. That is, lncRNA MEG3 can modulate the osteogenic differentiation of DPSCs through the miR-543/SMURF1/RUNX2 axis.

### 5.7. The Regulatory Role of LINC01133 in the Osteogenic Differentiation of DPSCs

LINC01133 is a gene interval lncRNA located on the long arm of region 2 of chromosome 1, with a highly conserved sequence. It has been found to play a role as an oncogene or an antioncogene in different cancers through various mechanisms and become a regulatory factor for cancers of the digestive, reproductive, urinary, respiratory, and skeletal systems. It is a potential marker for cancer prognosis [87]. LINC01133 promotes osteogenic differentiation of ASCs and PDLSCs, but in one study it was suggested that LINC01133 inhibited osteogenic differentiation of DPSCs. A reduction in osteogenic marker expression was observed when LINC01133 was overexpressed after osteo-differentiation of DPSCs. Further studies showed that LINC01133 could be used as a sponge for miR-199b-5p. Overexpression of LINC01133 up-regulated its downstream effector molecule AKT3 by secreting miR-199b-5p, which inhibited the osteogenic differentiation of DPSCs [88]. LINC01133 and the previously mentioned lncRNA MEG3, these lncRNAs show opposite effects on osteogenic differentiation in different MSCs, but since related studies are relatively rare, more studies are needed to make the role of these lncRNAs in regulating different MSCs more convincing.

### 5.8. The Regulatory Role of lncRNA ANCR in the Osteogenic Differentiation of DPSCs

Antidifferentiation noncoding RNA (ANCR), also known as differentiation antagonizing nonprotein coding RNA (DANCR) or small nucleolar RNA host gene protein 13 (SNHG13), is a newly discovered lncRNA whose expression decreases during stem cell differentiation. It has 855 base pairs and is located on human chromosome 4q12 [89]. lncRNA ANCR can promote the invasion and migration of glioma [90], breast cancer [91], and other diseases, but it can alleviate the progression of hepatocellular carcinoma [92]. There is evidence that lncRNA ANCR has important biological functions in osteogenesis. Down-regulation of ANCR can promote the differentiation of osteoblasts by interacting with EZH2 and then regulating the expression of RUNX2, indicating that ANCR is an important mediator of osteoblast differentiation [47]. In DPSCs, the loss-of-function assay of ANCR increased ALP activity, secreted a more mineralized matrix, and enhanced the expression of osteogenesis-related genes, including ALP, bone sialoprotein (BSP), and osteocalcin (OCN) [93].

### 5.9. The Regulatory Role of lncRNA CAAT1 in the Osteogenic Differentiation of DPSCs

The expression of lncRNA colon cancer-associated transcript 1 (CCAT1) increased significantly during the proliferation and differentiation of DPSCs. Moreover, during the differentiation of CCAT1-overexpressing DPSCs, the expressions of COL1, osteopontin (OPN), and OCN were significantly increased. CCAT1 directly binds to miR-218, which promotes the osteogenesis and proliferation of DPSCs. However, what specific signal axes they use to achieve regulation is still unknown [94].

## 6. lncRNAs That Regulate Osteogenic Differentiation of PDLSCs

**Figure 4:**
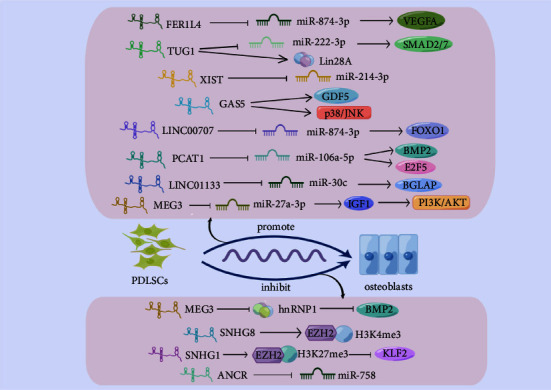
Mechanism of lncRNAs regulating osteogenic differentiation of PDLSCs by Figdraw (http://www.figdraw.com).

In osteogenic differentiation of PDLSCs, Gu et al. identified 960 lncRNAs that showed differential expression through RNA sequencing. Among them, 147 lncRNAs were predicted to bind to common miRNAs, indicating that specific lncRNAs may function as ceRNA in the osteogenic differentiation of PDLSCs [95]. The inflammatory environment can inhibit the osteo-differentiation of PDLSCs to a certain extent. Compared with unstimulated PDLSCs, the osteogenic capacity of PDLSCs was significantly reduced under the stimulation of TNF-*α*. The inflammatory environment has a certain impact on the expression of lncRNAs. 149 lncRNAs were up-regulated, and 169 lncRNAs were down-regulated during the osteogenic induction in the inflammatory microenvironment induced by TNF-*α*, which revealed that many lncRNAs play a role by acting as ceRNA to regulate transcripts [96]. Stem cells often live in the environment of tissue hypoxia, affecting the differentiation ability of stem cells. Scholars found that the osteoinduction of PDLSCs was inhibited under hypoxia. RNA sequencing was carried out for the expression difference of lncRNAs in PDLSCs under 20% oxygen concentration aerobic condition and 2% oxygen concentration hypoxia condition. According to the results, there was a significant change in the expression profile of lncRNAs. There were 449 lncRNAs with different expressions. Kyoto Encyclopedia of Genes and Genomes (KEGG) enrichment analysis found that the TGF-*β* signaling pathway is the key pathway that regulates the osteogenic differentiation of PDLSCs under hypoxia. Still, its regulatory pathway is worthy of specific exploration [97]. The osteogenesis mechanism of PDLSCs regulated by lncRNA can be seen in Figure 4.

### 6.1. The Regulatory Role of lncRNA FER1L4 in the Osteogenic Differentiation of PDLSCs

Fer-1-like family member 4 (FER1L4) is a lncRNA located on human chromosome 20 with a length of 6717 bp and is highly expressed in tissues. In various tumors, it plays a role in the occurrence and development, and cells in cancer can be inhibited from proliferating and migrating, such as esophageal squamous cells, carcinoma cells [98], hepatocellular carcinoma cells [99], and endometrial cancer cells [100]. But can promote the invasion and targeting of papillary thyroid cancer [101] and the progression of oral squamous cell carcinoma [102]. lncRNA FER1L4 has a little study on bone diseases, but some scholars have found that it can be a positive regulator of osteogenic differentiation in PDLSCs by targeting miR-874-3p/VEGFA. During the osteogenic induction of PDLSCs, the expression of lncRNA FER1L4 increased. FER1L4 has direct interaction sites with miR-874-3p, which can act as its sponge in osteogenic differentiation. Cotransfection showed that miR-874-3p partially reversed the osteogenic differentiation promotion of FER1L4. The 3′-UTR of VEGFA contains miR-874-3p binding sites, and overexpression of FER1L4 enhances VEGFA. PDLSCs and poly-lactic-co-glycolic acid (PLGA) scaffolds were loaded with and without FER1L4, respectively, and implanted into the cranial defects of nude mice. There was a greater amount of new bone formation in the FER1L4-overexpression group after 3D reconstruction [13].

### 6.2. The Regulatory Role of lncRNA TUG1 in the Osteogenic Differentiation of PDLSCs

TUG1 is a highly conserved lncRNA with a 7.1 kb length. It is also considered a potential tumor regulator. Many papers showed that it could enhance the development of epithelial ovarian cancer [103], cervical cancer [104], esophageal cancer [105], and other tumors. In addition, TUG1 plays an essential role in cardiovascular disease, which can aggravate ischemic myocardial injury [106] and promote atherosclerosis [107]. In calcified aortic valve disease (CAVD), lncRNA TUG1 interacts with miR-204-5p to increase the expression of RUNX2 to promote osteogenic differentiation, which may be the pathogenesis of CAVD [108]. In regulating the osteogenic differentiation of other cells, lncRNA TUG1 also showed certain functions. lncRNA TUG1 in osteoblasts can promote proliferation and differentiation by activating Wnt/*β*-catenin [109]. It can also reduce the expression of basic fibroblast growth factor (bFGF) protein through the ubiquitination of bFGF and promote the osteogenic differentiation of tendon stem cells [110].

TUG1 plays a ceRNA mechanism to participate in the regulation of PDLSCs osteogenic differentiation. As PDLSCs differentiate into osteogenic cells, the high expression of TUG1, including RUNX2, ALP, and OCN, encourages the expression of osteogenesis-related markers. In in-depth research of the mechanism, TUG1 acts as a sponge molecule for miRNA-222-3p to regulate osteogenic differentiation, and miR-222-3p targets small mother against decapentaplegic2/7(SMAD2/7) to promote the expression of osteogenesis-related genes of PDLSCs, and members of the Smad family can mediate the signal transduction of the TGF-*β* family. Many reports have shown the relationship between the Smad family and osteogenesis [111, 112]. Therefore, it is considered that the regulation of lncRNA TUG1/microRNA-222-3p/Smad2/7 can be a therapeutic point for repairing bone defects with PDLSCs [113]. Park et al. found that TUG1 may have multiple binding sites with Lin28A. Lin28A was found to promote the osteogenesis of human periosteum-derived cells [114]. In PDLSCs, Lin28A expression was significantly decreased after TUG1 inhibition, and ALP, OCN, and RUNX2 expressions were reduced in Lin28A inhibition assays. TUG1 can interact with Lin28A to promote osteogenic differentiation [39].

### 6.3. The Regulatory Role of lncRNA XIST in the Osteogenic Differentiation of PDLSCs

LncRNA XIST (X inactive specific transcript) is located on human chromosome Xq13.2 and is the product of the gene XIST. It is evident that XIST is closely related to tumors and affects the process and prognosis of tumors. However, it plays opposite roles in the regulation of different tumors. It can boost the occurrence and development of bladder cancer [115], gastric cancer [116], nasopharyngeal cancer [117], oral squamous cell carcinoma [118], and other cancers. But it can also act as a tumor suppressor in cervical cancer [119], hepatocellular carcinoma [120], and nonsmall cell lung cancer [121]. The expression of lncRNA XIST was confirmed to change during the induction of osteogenic differentiation of BMSCs. Zheng et al. found that the overexpression of XIST can increase ALP expression, while XIST as a knockout produced the opposite phenomenon. Mechanistically, XIST can target miR-9-5p to regulate ALP and promote osteogenic differentiation of BMSCs [122]. XIST also regulates the osteogenesis of PDLSCs in a significant way. A direct combination between XIST and miR-214-3p can play a role in the sponge adsorption of miR-214-3p in vitro. But XIST targeting miR-214 in regulating PDLSCs osteogenic differentiation-mediated possible signaling pathways or target genes needs further clarification [123].

### 6.4. The Regulatory Role of lncRNA GAS5 in the Osteogenic Differentiation of PDLSCs

Growth arrest specific 5 (GAS5) is located on chromosome 1q25 with a length of 650 nucleotides and is regarded as a potent tumor suppressor whose expression is associated with various cancers. It plays a regulatory role by acting as a sponge molecule of miRNA and epigenetic modifications and attenuates colorectal cancer [124], breast cancer [125], gastric cancer [126], etc. However, there are different views on its role in liver cancer. It has been reported that GAS5 can attenuate the invasion and migration of liver cancer cells [127]. GAS5 can also influence the osteogenic differentiation of stem cells. In the study of regulating the osteo-differentiation of BMSCs, lncRNA GAS5 was found to promote this process [128, 129]. It also decreased the osteogenesis of human vascular smooth muscle cells by modulating the GAS5/miR-26-5p/PTEN axis, thereby reducing the occurrence of vascular calcification [130]. Similarly, GAS5 also changed the progression of the osteogenic induction of PDLSCs. Loss- and gain-of-function experiments demonstrated that GAS5 enhanced the osteogenic induction of PDLSCs. The expression of growth differentiation factor 5 (GDF5) was increased in this process. GDF5 is alternatively known as BMP-14. Growth differentiation factors belong to a highly conserved subfamily of bone morphogenetic protein signaling molecules and are a relatively special member of the BMP family due to their structural and amino acid sequence characteristics. Its abnormal expression is associated with the occurrence and development of many bone diseases, including osteoarthritis [131] and rheumatoid arthritis [132]. GAS5 also increased the phosphorylation levels of JNK and p38 to partly alter the progressing of osteogenic differentiation of PDLSCs [133].

### 6.5. The Regulatory Role of LINC00707 in the Osteogenic Differentiation of PDLSCs

LINC00707 is an intergenic lncRNA located on 10p14. We found that LINC00707 can promote osteogenic differentiation of BMSCs by regulating WNT2B through competitive adsorption of miR-370-3p [134]. It has been found that LINC00707 can also be used for different targets to promote osteogenic differentiation of PDLSCs through similar mechanisms. LINC00707 directly binds to miRNA-490-3p. MiRNA-490-3p has a specific binding to forkhead box O1(FOXO1). FOXO1 targets miR-490-3p to alleviate cardiomyocyte damage [135] and inhibits osteogenic differentiation in thoracic ligamentum flavum cells [136]. FOXO1 is a member of the forkhead box O (FoxO) family and is related to bone diseases and osteogenic differentiation in multiple studies [137]. The results showed that LINC00707 and FOXO1 could promote the osteo-differentiation of PDLSCs, and the promotion of LINC00707 on the osteo-differentiation of PDLSCs was inhibited after miR-490-3p specifically combined with LINC00707. Further research found that LINC00707 could improve the expression of FOXO1 by sponging of miR-490-3p, and the LINC00707/miR-490-3p/FOXO1 axis could be used as a targeted treatment to regulate the osteogenesis of PDLSCs for bone regeneration [138].

### 6.6. The Regulatory Role of lncRNA PCAT1 in the Osteogenic Differentiation of PDLSCs

Prostate cancer-associated transcript 1 (PCAT1) was first discovered in prostate tissues and localized to chromosome 8q24. It is an intergenic lncRNA located in SNPs and near the c-MYC gene, which is involved in prostate cancer progression [139]. It also has a particular regulatory role in other specific cancers and is associated with prognosis [140, 141]. It may be safely said that various cancers are diagnosed and prognosis using lncRNA-PCAT1 as a marker. PCAT1 can sponge miRNA to regulate the osteogenesis of stem cells. Some scholars have found that PCAT1 can act as a ceRNA of miR-145-5p to induce osteogenic differentiation of ASCs by upregulating toll-like receptor (TLR4) expression and activating the TLR signaling pathway [142].

In addition, PCAT1 has been discussed as being involved in the osteogenic differentiation of PDLSCs. Jia et al. found that lncRNA PCAT1 interacts with miR-106a-5p to form lncRNA PCAT1/miR-106a-5p regulatory network. BMP2 is a critical gene that promotes the differentiation of MSCs into osteoblasts. lncRNA-PCAT1 can regulate the expression of BMP2 through sponge absorption of miR-106a-5p, thereby affecting the osteogenic differentiation of PDLSCs. In addition, they also found that the promoter of lncRNA PCAT1 can bind to another target of miR-106a-5p, E2F transcription factor 5 (E2F5). lncRNA PCAT1/miR-106a-5p can also regulate the process of E2F5, affecting the osteoinduction of PDLSCs. Changes in the expression of E2F5 could also affect the expression of lncRNA PCAT1 and miR-106a-5p, suggesting that lncRNA PCAT1/miR-106a-5p/E2F5 may regulate the osteoinduction of PDLSCs in a feed-forward regulation [143]. However, other reports have not reported the effect and specific mechanism of E2F5 on stem cell osteogenesis, and it is worthy of further study.

### 6.7. The Regulatory Role of lncRNA ANCR in the Osteogenic Differentiation of PDLSCs

The previously mentioned lncRNA ANCR not only plays a role in the osteogenesis of DPSCs and biological functions of cells and mediates the osteo-differentiation of PDLSCs. It has been reported that the expression of lncRNA ANCR is down-regulated during the osteo-differentiation of PDLSCs. Reducing the expression of ANCR can inhibit the proliferation of PDLSCs but promote their osteoinduction, and the mechanism of its regulation on osteogenesis has not been studied in depth in this research [144]. However, previous studies by Jia et al. have shown that reducing lncRNA ANCR promotes the proliferation and osteogenic differentiation of PDLSCs, which may be achieved by regulating the Wnt pathway [145]. Peng et al. reported that the inflammatory environment also made a difference in the osteogenesis of PDLSCs. The osteogenic capacity of PDLSCs extracted from periodontitis patients was lower than that of normal PDLSCs. They then explored the mechanism of lncRNA ANCR in regulating the osteogenic differentiation of PDLSCs in an inflammatory environment. lncRNA ANCR repressed the osteogenic process of PDLSCs. MiR-758 is the direct binding target of lncRNA ANCR and affects the osteogenic induction process of PDLSCs through its sponge effect. Further research shows that Notch2 binds to miR-758, and miR-758 inhibits Notch2-Wnt/*β*-catenin signaling pathway [146]. However, this study has not verified the regulation of Notch2 by lncRNA targeting miR-758 to regulate the osteogenic differentiation of PDLSCs. lncRNA ANCR can also serve as an important part of regulating the osteo-differentiation of PDLSCs. Its specific mechanism needs more research to explore.

### 6.8. The Regulatory Role of LINC01133 in the Osteogenic Differentiation of PDLSCs

Unlike the regulatory effect on DPSCs, LINC01133 promoted the osteo-differentiation of PDLSCs. Compared with tissues in periodontitis, the expression of LINC01133 in periodontitis was lower. LINC01133 inhibits the expression of miR-30c by interacting with miR-30c via the ceRNA network. MiR-30c can specifically bind to bone gamma carboxyglutamate protein (BGLAP), a small molecule protein released by osteoblasts, which can encode OCN [147]. Knockdown BGLAP can reverse the effect of miR-30c reduction on the osteoinduction of PDLSCs. The mechanism of LINC01133/miR-30c/BGLAP regulating the osteogenesis of PDLSCs may be involved in periodontitis [148].

### 6.9. The Regulatory Role of lncRNA MEG3 in the Osteogenic Differentiation of PDLSCs

The expression of lncRNA MEG3 decreased in osteo-differentiation of periodontal ligament cells. The overexpression of MEG3 reduced the expression of BMP2 and hnRNPI. hnRNPI is an RBP with multiple RNA binding domains, and MEG3 may inhibit the expression of BMP2 through its interaction with hnRNPI, thus affecting the osteo-differentiation of periodontal ligament cells [40]. Comparing healthy periodontal tissues and genes, mRNA IGF1 and lncRNA MEG3 in the PI3K/Akt signaling pathway were differentially expressed in periodontitis and healthy periodontal tissues. IGF1 is considered a protein that the osteogenic differentiation of PDLSCs can be promoted [149]. By constructing the network diagram, it was found that MEG3 was positively correlated with IGF1. It was further predicted that miR-27a-3p could act as a bridge between the two, and MEG3 expression was positively correlated with the expression levels of osteogenic differentiation markers (including RUNX2, Osterix, OCN, and Colla1). Further studies on its mechanism showed that MEG3 could regulate the osteogenic differentiation of PDLSCs through miR-27a-3p, and miR-27a-3p could regulate the osteogenic differentiation through IGF1. Using the PI3K inhibitor LY294002 inhibited osteo-differentiation. It can be concluded that the osteogenic performance can be boosted via lncRNA MEG3/miR-27a-3p/IGF1 axis activated by the PI3K/Akt signaling pathway [150].

### 6.10. The Regulatory Role of lncRNA HHIP-AS1 in the Osteogenic Differentiation of PDLSCs

lncRNA hedgehog-interacting protein antisense RNA 1 (HHIP-AS1), located on the long arm of chromosome 4, inhibits the proliferation and invasion of hepatocellular carcinoma cells and promotes apoptosis [151]. The osteoinduction of PDLSCs was inhibited by continuous compressive stress. There was downregulation of the lncRNA HHIP-AS1 in the process. Further studies found that knockdown HHIP-AS1 inhibited the expression of bone-related biomarkers during the osteo-differentiation of PDLSCs, while overexpression of HHIP-AS1 showed the opposite result. lncRNA HHIP-AS1 can be applied to orthodontic treatment as a target to accelerate tooth movement. At the same time, the RNA sequencing results showed that 356 mRNA expressions were increased and 185 expressions were down-regulated in HHIP-AS1 deleted PDLSCs. Bioinformatics analysis results revealed that signaling pathways such as PI3K/AKT and JAK/STAT were related to HHIP-AS1 function. However, the specific mechanism of HHIP-AS1 regulating the osteo-differentiation of PDLSCs under stress needs to be further revealed [152].

### 6.11. The Regulatory Role of lncRNA SNHG1 in the Osteogenic Differentiation of PDLSCs

In addition to the classical mechanism of lncRNA as ceRNA, some scholars have also found that lncRNA can regulate the osteogenic differentiation process of PDLSCs through epigenetic modification. Small nucleolar RNA host gene 1 (SNHG1), a kind of lncRNA located at 11q12.3, interacts with EZH2 in many tumors to influence disease progression. High expression of SNHG1 in patients with colorectal cancer can interact with EZH2 to regulate histone methylation of Krüppel-like factor 2 (KLF2) and cyclin-dependent kinase inhibitor 2B (CDKN2B) in the nucleus and affect the biological behavior of colorectal cancer cells [153]. Combination with EZH2 inhibits the transcription of CDKN1A and CDKN2B, thereby promoting hepatocellular carcinoma development [154]. lncRNA SNHG1 impacts the osteogenesis of BMSCs. It can repress Wnt/*β*-catenin signaling pathway through MiR-101/DKK1 (Dickkopf-1) axis during the osteogenesis [155]. And it can also attenuate p38 MAPK signaling through Nedd4 ubiquitination during osteogenic induction. It may be safely said that SNHG1 is a negative regulator of osteogenesis in BMSCs [156].

lncRNA SNHG1 also plays a similar role in PDLSCs' osteogenic performance. In the osteogenesis of PDLSCs, the expression of SNHG1 was down-regulated, and the expression of KLF2 was increased. Mechanistic investigations demonstrated its interaction with EZH2 to silence KLF2 expression by methylating promoter histones, thereby inhibiting osteogenesis. KLF2 is a transcription factor containing a highly conserved DNA-binding zinc finger domain that targets RUNX2 to regulate osteoblast differentiation and becomes a significant regulator of osteogenic differentiation [157]. lncRNA SNHG1/EZH2/KLF2 axis can be a therapeutic target for regulating the osteo-differentiation of PDLSCs and bone regeneration [158].

### 6.12. The Regulatory Role of lncRNA SNHG8 in the Osteogenic Differentiation of PDLSC

Small nucleolar RNA host gene 8 (SNHG8) is a mechanical force-sensitive lncRNA whose expression is significantly lower in PDLSCs subjected to mechanical strength than in stem cells without stress [159]. A reduction in SNHG8 expression promotes osteo-differentiation of PDLSCs. Experiments in vivo have also shown the key role of SNHG8 in regulating the osteo-differentiation of PDLSCs. During the tooth movement of rats, the expression of small integral membrane protein 4(SMIM4), the homologous gene of SNHG8, decreased in the early stage. PDLSCs interfered with SNHG8 also showed more powerful ectopic osteogenic ability in nude mice. Mechanistically, the nucleus is a major location for SNHG8, and there is an interaction between SNHG8 and EZH2. They also found that after 24 h of mechanical force, the trimethylation of histone H3 at lysine 4 (H3K4me3) level in the promoter region of SNHG8 and 250 bp upstream of the promoter decreased, which may affect the transcription level of SNHG8 and may be an important way for lncRNA SNHG8 to regulate PDLSCs at the epigenetic level under mechanical force [160].

## 7. lncRNAs That Regulate Osteogenic Differentiation of SCAPs

Some scholars have found that the periapical papilla contains many MSCs, a new group of pluripotent stem cells called stem cells from the apical papilla (SCAPs). It has been shown that SCAPs have a greater ability to form mineralized matrix compared to DPSCs from the same tooth, which can be considered an advantage for SCAPs as being the seed cells for future bone tissue engineering [161].

### 7.1. The Regulatory Role of lncRNA H19 in the Osteogenic Differentiation of SCAPs

**Figure 5:**
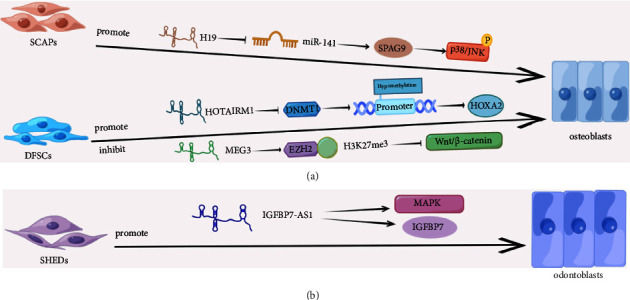
(a) Mechanism of lncRNAs regulating osteogenic differentiation of SCAPs and DFSCs. (b) Mechanism of lncRNAs regulating odontogenic differentiation of SHEDs by Figdraw (http://www.figdraw.com).

H19 is located on chromosome 11p15.5, and the length is 2.7 kb, expressed by the maternal gene. It serves an important role in tumorigenesis and may contribute to cancer development in humans through ceRNA mechanisms, methylation, and other means. It can promote the progression of various cancers, including breast cancer [162], colorectal cancer [163], and lung cancer [164], but can inhibit the development of papillary thyroid cancer [165]. H19 is also reported to be associated with stemness. Some studies have been carried out on the regulation of osteogenesis by H19. lncRNA H19 can promote the matrix mineralization of osteoblasts [166] and play an essential role in the osteo-differentiation of BMSCs [167]. The overexpression of H19 leads to the escalation of the expression of markers associated with osteogenesis in SCAPs, and experiments in vivo also proved it. MiR-141 can competitively bind to H19 and regulate the osteogenic performance of SCAPs negatively. MiR-141 can also target sperm-associated antigen 9 (SPAG9) and degrade the expression of SPAG9. In other words, H19 can act as a sponge of miR-141, prevent SPAG9 from being degraded, participate in the MAPK pathway by modulating JNK and p38 phosphorylation, and promote the continuous osteogenic differentiation of SCAPs. Therefore, lncRNA H19/miR-141/SPAG9 axis can also be used as an important target to regulate the osteogenic differentiation of SCAPs [168] (Figure 5(a)).

### 7.2. The Regulatory Role of lncRNA ANCR in the Osteogenic Differentiation of SCAPs

There was a research on SCAPs reported that downregulation of lncRNA ANCR had no significant effect on SCAPs proliferation but promoted its osteogenic differentiation. In addition, down-regulation of lncRNA ANCR promotes SCAPs adipogenic differentiation and neural differentiation, so it can be considered that lncRNA ANCR is an important regulator of SCAPs proliferation and differentiation [93].

## 8. lncRNAs That Regulate Osteogenic Differentiation of DFSCs

The dental follicle comes from the neural crest, which is the connective tissue sac around the enamel and dental papilla during the development of tooth germ [8]. It is mainly composed of ectodermal mesenchymal dental sac cells, in which stem cells also exist. Cells in the dental follicle can develop into periodontal tissues such as cementum, periodontal ligament, and alveolar bone, so DFSCs are also considered the precursors of PDLSCs. DFSCs have the potential for multidirectional differentiation and express more stemness-related genes than PDLSCs, indicating that DFSCs are more pluripotent than PDLSCs. In addition, DFSCs are easy to culture and have a stronger ability to proliferate and form colonies than DPSCs [169]. DFSCs can be induced to differentiate into osteoblasts [170], and DFSCs have stronger osteogenic ability than skin and bone marrow-derived MSCs *in vivo* [171]. All of these indicate that DFSCs have potential and application prospects in the treatment of craniomaxillofacial and periodontal bone defects. The osteogenesis mechanism of DFSCs regulated by lncRNA can be seen in Figure 5(a).

### 8.1. The Regulatory Role of lncRNA HOTARM1 in the Osteogenic Differentiation of DFSCs

HOXA transcript antisense RNA, myeloid-specific 1 (HOTAIRM1) is an antisense transcript with transcriptional activity between HOXA1 and HOXA2 genes, which is specifically expressed in bone marrow lines [172]. HOXA2 is a target of HOTAIRM1, which is needed in osteogenesis [173]. Many studies have shown that lncRNA HOTAIRM1 can be a molecular target in the regulation of osteogenesis. It can promote osteoblast differentiation by inhibiting the NF-*κ*B pathway [174]. It can also regulate the JNK/AP-1 signaling pathway through epigenetic modification to regulate the transcription of RUNX2 and promote the osteogenic differentiation of BMSCs [58]. Chen et al. found that lncRNA HOTAIRM1 and HOXA2 were more expressed in DFSCs than PDLSCs. In addition, HOTAIRM1 and HOXA2 were similarly expressed in dental follicles and periodontal tissues during mouse tooth development. Mechanistically, they reported that HOTAIRM1 repressed the expression of DNMT1 and the enrichment of DNMT1 on the HOXA2 promoter, making hypomethylation of the promoter, and lncRNA HOTAIRM1-mediated demethylation can activate HOXA2 transcription, thereby promoting the in vitro osteoinduction of DFSCs [175].

### 8.2. The Regulatory Role of lncRNA MEG3 in the Osteogenic Differentiation of DFSCs

LncRNA MEG3 can regulate the osteogenic differentiation of DFSCs through epigenetic modification. Compared with PDLSCs, lnRNAMEG3 was significantly down-regulated in DFSCs. lncRNA MEG3 increased the expression of H3K27me3 through interaction with EZH2. H3K27me3 could enrich the promoter of Wnt ligand and inhibit Wnt/*β*-catenin. Down-regulated lncRNA MEG3 enhances osteo-differentiation of DFSCs by an epigenetically modulated Wnt pathway [176]. Therefore, osteogenic differentiation in DFSCs can be promoted by downregulating the expression of lncRNA MEG3.

## 9. lncRNAs That Regulate Osteogenic Differentiation of GMSCs

In 2009, Zhang et al. first isolated a new stem cell population from human gingiva—GMSCs, with high differentiation and colony-forming ability in vitro. They can also differentiate into different cell lines, including osteogenic differentiation, adipogenic differentiation, and neural differentiation. GMSCs also possess the stem cell properties of self-renewal and differentiation ability *in vivo* [7]. Using microarray analysis, Jia et al. examined the expression profiles of lncRNAs and mRNAs in PDLSCs and GMSCs. They found that 2162 lncRNAs were significantly different in expression between these two stem cell populations, and 735 lncRNAs were highly expressed in PDLSCs and 1427 in GMSCs. The functions of most of the differentially expressed lncRNAs are unknown [177]. Afterward, the research group analyzed the key lncRNAs and mRNAs during the osteogenic differentiation of PDLSCs and GMSCs. The expressions of 238 lncRNAs were only changed in the osteogenic differentiation of PDLSCs, while the number of GMSCs was 170. The difference between the two may be the basis of the osteogenic differentiation potential of the two kinds of stem cells. However, 126 lncRNAs were found to have similar expression changes in the osteo-differentiation of PDLSCs and GMSCs. They also found that DKK1 (the inhibitor of the Wnt classical pathway) could attenuate the osteogenic differentiation ability of GMSCs and found that lncRNA ENST00000365271, n407948, TCONS_00018416-XLOC_008700, and n334561 showed positively correlated DKK1 expression, while n334022 showed negatively correlated with the expression of DKK1. Whether lncRNA can regulate the osteogenic differentiation of GMSCs by regulating DKK1 must be further explored [178].

## 10. lncRNAs That Regulate Osteogenic Differentiation of SHEDs

SHEDs were first discovered in 2003 when Miura et al. isolated deciduous DPSCs from human deciduous teeth. It is a stem cell population that can proliferate and differentiate pluripotently and has stronger proliferative and osteoinductive abilities than DPSCs. They found that SHEDs do not directly differentiate into osteoblasts but form a template to induce their osteoblasts to form new bone [10]. But other scholars had found something different: when SHEDs with hydroxyapatite/tricalcium phosphate as a carrier were used to repair calvarial defects in immunodeficient mice, continuous bone formation was observed in the defect area; however, SHED-mediated bone formation lacks the hematopoietic bone marrow components common in BMSCs [179]. This study indicates that SHEDs can differentiate into osteoblasts, which differs from Miura et al.'s induction of host cells to form bone structures [10].

Osteogenic differentiation and odontogenic differentiation exhibit numerous similarities, as evidenced by their shared expression of common biomarkers during differentiation, including ALP, BMP, BSP, and OCN. However, a notable distinction between the two processes is the requirement for specific transcription factors, such as dentin sialophosphate protein (DSPP), which are critical for dentin formation during odontogenic differentiation but are not expressed in cells undergoing osteogenic differentiation. High-throughput sequencing analyzed the expression levels of lncRNAs during SHEDs odonto-differentiation. After 7 days, 1138 lncRNAs were differentially expressed (569 up-regulated, 569 down-regulated), and 1358 lncRNAs were differentially expressed on day 14 (767 up-regulated, 591 down-regulated). The expression of lncRNA IGFBP7-AS1 increased during SHEDs' odontogenic induction. When IGFBP7-AS1 was overexpressed, ALP and mineralized matrix levels increased, while knockdown IGFBP7-AS1 showed the opposite phenomenon. Knockdown of IGFBP7-AS1 also significantly reduced p-p38/p38 levels. ALP activity and alizarin red staining were inhibited when the p38 signaling pathway inhibitor SB203580 was used, indicating that lncRNA IGFBP7-AS1 odonto-differentiation of SHEDs by activating MAPK signaling pathway [180]. The follow-up results also found a positive correlation between lncRNA IGFBP7-AS1 and IGFBP7 in the expression of SHEDs' odontogenic differentiation. lncRNA IGFBP7-AS1 increases the stability of IGFBP7 and may be a target to promote SHEDs' odontogenic differentiation [181] (Figure 5(b)).

## 11. Conclusions and Future Perspectives

Whether *in vivo* or *in vitro*, different types of MSCs from dental tissues have differences in osteogenic differentiation. Still, they have strong osteogenic differentiation potential and are easy to obtain, which is considered able to replace BMSCs as an alternative clinical therapy. lncRNAs use their sequences and structures to interact with RNA, DNA, and proteins to play biological roles.

Although some lncRNAs regulating the osteogenic differentiation of stem cells from dental tissues have been identified from the available studies, some of the mechanisms of action have been uncovered. However, most of these studies have focused on the interaction of lncRNAs with miRNAs, and studies on the role of lncRNAs combined with DNA and protein are rare. So expanding the role of lncRNAs in the osteogenic differentiation of stem cells is an important research direction in the future to screen the lncRNA that regulates the osteogenesis of odontogenic stem cells and further studies its key mechanisms. Furthermore, with the widespread use of high-throughput sequencing, a large amount of lncRNA-related data have been generated, but the analysis of these data remains challenging, and more effective methods need to be developed to reveal the regulatory role of lncRNAs in stem cells from dental tissues. lncRNA could become a new regulator for stem cell therapy to repair bone defects. Unfortunately, current research on lncRNA as a target for bone regeneration stops at animal experiments. In the future, it is important to correlate lncRNA with relevant bone diseases treated using stem cell osteogenic differentiation to develop new therapeutic modalities with the aim of improving treatment outcomes. There is still a long way to go before lncRNA can be transformed into clinical trials.

## Data Availability

The data supporting the current study are available from the corresponding author upon request.
